# Long-Term Effects of Methadone Maintenance Treatment with Different Psychosocial Intervention Models

**DOI:** 10.1371/journal.pone.0087931

**Published:** 2014-02-03

**Authors:** Lirong Wang, Xiaoli Wei, Xueliang Wang, Jinsong Li, Hengxin Li, Wei Jia

**Affiliations:** 1 Department of Epidemiology and Biostatistics, School of Public Health, Xi'an Jiaotong University Health Science Center, Xi'an, Shaanxi, China; 2 Xi'an Center for Disease Control and Prevention, Xi'an, Shaanxi, China; 3 Methadone Maintenance Therapy Clinic, Xi’an Mental Health Center, Xi'an, Shaanxi, China; Temple University School of Medicine, United States of America

## Abstract

This study evaluated the long-term effects of different psychosocial intervention models in methadone maintenance treatment (MMT) in Xi'an China. Patients from five MMT clinics were divided into three groups receiving MMT only, MMT with counseling psychology (CP) or MMT with contingency management (CM). A five-year follow-up was carried out with daily records of medication, monthly random urine morphine tests, and tests for anti-HIV and anti-HCV every six months. Drug use behavior was recorded six months after initial recruitment using a survey. Adjusted *RRs* and their 95% confidence intervals (*CI*s) were estimated using an unconditional logistic regression model or a Cox proportional hazard model. A total of 2662 patients were recruited with 797 in MMT, 985 in MMT with CP, and 880 in MMT with CM. Following six months of treatment, the injection rates of MMT with CP and MMT with CM groups were significantly lower than that of MMT (5.1% and 6.9% vs. 16.3%, *x^2^*  =  47.093 and 29.908, respectively; *P*<0.05). HIV incidences for MMT, MMT with CP and MMT with CM at the five year follow-up were 20.09, 0.00 and 10.02 per ten thousand person-years, respectively. HCV incidences were 18.35, 4.42 and 6.61 per hundred person-years, respectively, demonstrating that CP and CM were protective factors for HCV incidence (*RR*  =  0.209 and 0.414, with range of 0.146 – 0.300 and 0.298 – 0.574, respectively). MMT supplemented with CP or CM can reduce heroin use and related risk behaviors, thereby reducing the incidence of HIV and HCV.

## Introduction

Since the 1980s, drug abuse has become increasingly rampant in China with registered numbers of drug users increasing from 70,000 in 1990 to 1.794 million by the end of 2011 [1], [2]. Consequently, HIV and HCV transmission through drug use has also become an emergent problem [3]–[5]. By the end of 2011, the cumulative number of reported people living with HIV (PLHIV) was 445,000, including 174,000 AIDS cases and 93,000 recorded deaths. Furthermore, recent epidemic estimates suggest that 780,000 (between 620,000 and 940,000) people living in China are infected with HIV [6]. Among the cumulative number of PLHIV in China reported in 2007, 38.5% were infected via drug injection [7], with infection rates of 17.8%, 25.0% and 59.9%, in Guangxi, Sichuan and Yunnan, respectively [8]–[10]. Another report based on a systematic review of 40 studies from 1997 to 2007 showed that the prevalence of HIV infection was 12.6% and the HCV prevalence rate was 67.0% among injection drug users in China [11].

The Chinese government has noted the problems caused by drug use, including hazards for drug addicts themselves, various social problems and HIV infection. As a result, the government has launched a variety of measures to deal with drug use, such as voluntary and mandatory drug treatment, and labor rehabilitation after withdrawing from drug use [12], [13]. From March to June of 2004, eight methadone maintenance treatment (MMT) clinics were set up in five provinces (or autonomous regions), including Yunnan, Guizhou, Sichuan, Guangxi, and Zhejiang in China [14]. Subsequently, MMT developed rapidly in various parts of China [15]. By November 2012, 755 clinics had been set up in 28 provinces, autonomous regions, municipalities directly under the central government in China with more than 500 registered opioid addicts, treating nearly 0.21 million addicts, with a cumulative treatment number of 0.38 million. The average treatment number of each clinic was 277 with yearly treatment retention rates of 78.2% [16].

Studies have shown that MMT is beneficial in the control of drug abuse and prevention of HIV infection, and its main effects are reflected in the reduction of drug use behaviors, decreased high-risk sexual behaviors, reduced drug-related deaths, restoration of social and family functions, and a reduction in crime rate [17]–[21]. The one-year follow-up results from the first eight MMT clinics in China also showed similar effects [22]. However, Yin *et al.* [23] along with a meta-analysis study on the effect of interventions in MMT clinics in China by Xu *et al.* [24] reported high drop-out rates in MMT clinics with one-year retention rates below 60%, and widespread illicit drug use in outpatients. Some of the most important issues regarding MMT in China involve reducing the drop-out rate, increasing the retention rate, and promoting the return of drug addicts to society.

In Guangdong province, results from a survey of 300 newly enrolled drug abuse patients revealed a common misconception regarding MMT. The results showed that 92.3% of those surveyed thought methadone was a drug rehabilitation therapy, 64.2% thought addiction could be completely cured after two to three months of methadone use, 77.9% thought it was not necessary to continue lifelong methadone use, and 84.3% thought that methadone dosage needed to be gradually reduced to minimize harmful effects [24]. These general misconceptions may explain the high MMT drop-out rate. A recent study suggested that MMT treatment would be more effective when accompanied by counseling psychology (CP) and other social support services [25]. In addition to CP, contingency management (CM) is another way to improve the effect of MMT [26], and there have been studies comparing the reduction in illicit drug use with CM and CP following six months of intervention [27], [28]. A more recent randomized controlled trial conducted in five MMT clinics in Kunming and Shanghai observed that CM reduced illicit drug use and increased the retention in treatment after six months [29]. Chawarski *et al.* [30] performed behavioral drug and HIV risk reduction counseling (BDRC) interventions for three months in MMT clinics in Wuhan and followed patients for an additional six months. Their study showed that BDRC increased the MMT retention rate and reduced illicit drug use and behaviors that risk HIV infection. MMT programs have only recently been established in China, and therefore current studies have demonstrated only short-term effects, within six to 12 months following treatment [31], [32]. Furthermore, there are no studies examining the effect of CP and CM on long-term retention in MMT or the rate of HIV and HCV infection in China.

Xi'an is an inland city in China, which is a traffic hub connecting Yunnan, Sichuan, Xinjiang and other areas with high drug abuse. The first case of HIV infection in Xi'an was reported in 1992, and as of October 31, 2012, a cumulative 1574 HIV/AIDS cases have been reported [33]. A 2010 study reported that HIV prevalence among drug users in Xi'an was 1.3% and HCV prevalence was 60.0% [34]. In this study, patients from five MMT clinics in Xi'an were divided into three groups receiving MMT only, MMT with CP or MMT with CM. A five-year follow-up was carried out to evaluate the short-term and long-term effects of different psychosocial intervention on drug use behavior, MMT retention, and HIV/HCV incidence.

## Methods

### Ethics statement

The study was reviewed and approved by the Ethics Committee of Xi’an Jiaotong University. The written informed consent was obtained from each participant before the study.

### Participants

In 2006, there were a total of five MMT clinics in Xi'an City of China, including Weiyang Second People's Hospital, Beilin Second People's Hospital, First Outpatients Department of Mental Health Center, Second Outpatients Department of Mental Health Center, and the Chinese Medicine Hospital of Xincheng District. All drug users treated in these five clinics from July 1, 2006 to March 31, 2007 were recruited for the study. According to the current regulations, patients must meet the following criteria to be eligible for MMT: (1) opioid abuse addicts with unsuccessful detoxification after several treatments; (2) over 20 years of age; (3) residents of the counties (cities, districts) where MMT clinics are located, or registered citizens of a foreign household with local residence for at least six months and have a local temporary residence permit; (4) demonstrate full civil capacity. Participants with severe asthma, severe liver and kidney dysfunction who cannot participate in treatment were excluded from the study.

### Study process

Participants were divided into three groups according to the three outpatient management modes. Participants who went to the Weiyang Second People's Hospital and Beilin Second People's Hospital received MMT only; participants who went to First and Second Outpatients Department of Mental Health Centers received MMT with CP; and participants who went to the Chinese Medicine Hospital of Xincheng District received MMT with CM. A baseline survey was carried out to get personal and drug use information on the day the participant was included, and anti-HIV and anti-HCV tests were performed within one month of the recruitment.

In the MMT group, each day (or every other day for a small number of participants), the included participants needed to receive a prescribed amount of methadone oral solution at the clinic under a doctor’s supervision. Urine morphine tests were performed on all participants on a randomly selected day each month to check for illicit drug use. If urine tests could not be obtained, another day would be randomly selected to ensure that all participants were randomly checked at least once a month.

Participants in the MMT with CP group received similar oral methadone and monthly random urine morphine tests. However, these participants also received six months of psychological counseling (twice per month) in the form of group interviews or individual counseling by psychological practitioners, with the counseling sessions lasting for 15–20 minutes each time. The counseling was provided by trained psychological practitioners. Counseling methods and content were set and trained by Chinese Center for Disease Control and Prevention, which were arranged in six phases according to the different psychological stages of participating patients. The first phase of counseling provided an introduction pertaining to the number of days needed to achieve the best effect of MMT, consolidation principles on the side effects of early MMT, and information on the potential harm produced by the combined use of sedatives and alcohol. In the second phase, participants were counseled on the influence of social networks, including how to avoid contacts with heroin-taking friends and to resist the temptation to take drugs. In the third stage, treatment for drug dependence was equated to other medical treatments, the requirement for long-term methadone maintenance was discussed, and the advantages and disadvantages of treatment and withdrawal were introduced. The fourth stage involved role-playing exercises to demonstrate the supervisory and assistive role of family members. In the fifth stage, incentive measures were employed to strengthen the participants’ confidence. To reward treatment compliance, participants were awarded with small gifts (equal to approximately RMB10). Alternatively, the participant was required to provide reasons for non-compliance, and received guidance in return. The sixth and final phase involved the communication of treatment experience between doctors and participants. Each participant was required to sign name and treatment card number after each session to ensure that all six stages of psychological counseling were achieved.

Participants in the MMT with CM group also received daily oral methadone and monthly random urine morphine tests. These participants were enrolled in a six-month-long incentive program, with the incentive determined by the number of daily medications obtained (treatment compliance). Participants receiving more than 25 daily doses of methadone were given 3 doses free of charge (RMB30). Furthermore, random urine morphine tests were taken weekly to check for illicit drug use in the incentive program, participants with negative urine morphine test results were given bonus points (accumulation of three points could be exchanged for an RMB10 commodity, five points for an RMB20 commodity, ten points for an RMB50 commodity). Participants with positive urine tests received individual counseling, criticism, and were required to give written guarantees. Treatment eligibility was revoked after an accumulation of three positive test results.

A follow-up survey was performed following the six months of intervention to document changes in drug use behavior. Additionally, anti-HIV and anti-HCV tests were carried out every six months. Participants were followed-up until December 31, 2011.

### Laboratory tests

**Urinary morphine test.** Morphine test reagents (colloidal gold method) from Shanghai Rongsheng Biotechnology Co., Ltd. were used to detect urine morphine of participants.

**Anti-HIV test.** Primary screening for HIV infection was performed using an Anti-HIV1/2 EIA Kit (Sandwich) (Zhuhai Lizhu Diagnostics Inc.), and positive results were retested using a Diagnostic Kit for Antibodies to HIV Type 1 and/or 2 and HIV-1 Antigen (ELISA) (BioMérieux Corporate). Positive retest results were further confirmed using HIV-1+2 antibody immunoblotting reagents (HIVBLOT 2.2) (MP Biomedicals Asia Pacific Pte Ltd.).

**Anti-HCV test.** An Anti-HCV EIA Kit from Zhuhai Lizhu Diagnostics Inc. was used to detect anti-HCV. Participants positive for HCV antibodies were retested with an Anti-HCV ELISA KIT from Beijing Wantai Biological Co., Ltd.

### Data definitions

**Methadone dose.** The time-weighted average dosing of oral methadone (1 mg/ml) was calculated as follows: ml  =  Σ(dose, ml × number of days at that dose)/Σ(number of days receiving each dose).

**Residual period.** Urine test results obtained seven days before and after the first medication date comprised the residual period results. The analyses in the study excluded urine test results collected during the residual period, as these results were expected to be positive.

**Illicit drug use.** Illicit drug use was determined from the last urine test result. A positive result from the last urine test indicated illicit drug use by the participant.

**Drop-out (termination of treatment).** Before the end of the study (December 31, 2011), participants who did not go to an MMT clinic for medication for 30 consecutive days were regarded as drop-outs. Those who received the last medication before December 1, 2011 were designated as drop-outs, while participants who went to an MMT clinic for medication after December 1, 2011 were recorded as censored.

**Treatment retention time.** The treatment retention time was the time interval between the first medication date and the last medication date.

**Follow-up time of the anti-HIV/anti-HCV negative participant.** For participants without a seroconversion of anti-HIV/anti-HCV, the time interval between first medication date and the last medication date determined the follow-up time. For participants with anti-HIV/anti-HCV seroconversions, the time interval between the first medication date and the date of the seroconversion was designated as the follow-up time.

### Statistical analysis

Differences among groups were tested using a nonparametric two-tailed Mann-Whitney U test for continuous variables and a chi-squared (*x^2^*) statistic for categorical variables with Bonferroni's correction. Unconditional logistic regression models were used to analyze factors influencing changes in drug use behavior and illicit drug use. Life tables were used to calculate cumulative retention rates and to draw retention curves. Log-rank tests were used to compare retention rates between groups. Risk factors for MMT retention and HCV antibody seroconversion were analyzed with a Cox proportional hazard model. Risk factor assignments in an unconditional logistic regression model or a Cox proportional hazard model were shown in Table 1. Statistical significance was assessed using two-sided tests with α  =  0.05 for all analyses.

**Table 1:** Risk factor assignment in logistic and Cox regression analyses.

Factors		Value Assignment
Group	*X_1_*	MMT = 0, MMT with CP = 1, MMT with CM = 2
Gender	*X_2_*	Female = 0, Male = 1
Age (years)	*X_3_*	≤30 = 1, 31 – 40 = 2, > 40 = 3
Years of drug use	*X_4_*	≤5 = 1, 6 – 10 = 2, > 10 = 3
Ethnicity	*X_5_*	Han = 0, Other = 1
Education (years)	*X_6_*	≤9 = 0, >9 = 1
Marital status	*X_7_*	Single = 1, Married or have a regular partner = 2, Divorced, separated or widowed = 3
Employment	*X_8_*	No = 0, Yes = 1
Injecting	*X_9_*	No = 0, Yes = 1
Needle sharing	*X_10_*	No = 0, Yes = 1
Dosage (mg)	*X_11_*	≤30 = 1, 31 – 60 = 2, >60 = 3

## Results

### General characteristics

A total of 2758 participants were recruited from July 1, 2006 to March 31, 2007. Ninety-six participants were excluded for not meeting the criteria, including 42 in MMT, 31 in MMT with CP and 23 in MMT with CM. Therefore, 2662 participants were included in the study, with 797 participants in the MMT group, 985 in the MMT with CP group, and 880 in the MMT with CM group. No statistically significant differences were found between the three groups in age, gender, age of first drug use, years of drug use, or drug use by injection (Table 2).

**Table 2:** Demographic information of participants.

	MMT	MMT with CP	MMT with CM	Total
	(*n_1_* = 797)	(*n_2_* = 985)	(*n_3_* = 880)	(*n* = 2662)
Age in years, *M* (*SD*)	36.6 (5.99)	37.1 (5.82)	36.9 (5.85)	36.9 (5.88)
Age of first drug use, *M* (*SD*)	26.5 (6.83)	26.8 (6.29)	27.0 (7.08)	26.8 (6.72)
Years of drug use, *M* (*SD*)	9.7 (5.58)	9.9 (4.91)	9.4 (5.52)	9.7 (5.32)
Gender, *n* (%)				
Male	660 (82.8)	809 (82.1)	714 (81.1)	2183 (82.0)
Female	137 (17.2)	176 (17.9)	166 (18.9)	479 (18.0)
Ethnicity, *n* (%)				
Han	769 (96.5)^a^	845 (85.8)^b^	807 (91.7)^c^	2421 (90.9)
Other	28 (3.5)	140 (14.2)	73 (8.3)	241 (9.1)
Education, *n* (%)				
Elementary or lower	62 (7.8)	106 (10.8)^b^	71 (8.1)	239 (9.0)
Secondary school	453 (56.8)	535 (54.3)	452 (51.4)	1440 (54.1)
High school or higher	282 (35.4)	344 (34.9)	357 (40.6)	983 (36.9)
Marital status, *n* (%)				
Single	234 (29.4)^a^	332 (33.7)	303 (34.4)	869 (32.6)
Married or have a regular partner	426 (53.5)	549 (55.7)	452 (51.4)	1427 (53.6)
Divorced, separated or widowed	137 (17.2)	104 (10.6)	125 (14.2)	366 (13.7)
Employment, *n* (%)				
Yes	263 (33.0)^a^	177 (18.0)	177 (20.1)^c^	617 (23.2)
No	534 (67.0)	808 (82.0)	703 (79.9)	2045 (76.8)
Injecting, *n* (%)				
Yes	641 (80.4)	791 (80.3)	670 (76.1)	2102 (79.0)
No	156 (19.6)	194 (19.7)	210 (23.9)	560 (21.0)
Needle sharing, *n* (%)				
Yes	130 (16.3)^a^	466 (47.3)^b^	150 (17.0)	746 (28.0)
No	667 (83.7)	519 (52.7)	730 (83.0)	1916 (72.0)
Dosage (mg), *M* (*SD*)	46.3 (17.07)^a^	57.6 (19.54)^b^	49.6 (15.30)^c^	51.6 (18.13)

a MMT vs. MMT with CP, *P*<0.05.

b MMT with CP vs. MMT with CM, *P*<0.05.

c MMT vs. MMT with CM, *P*<0.05.

### Behavioral changes in drug addicts

At the six-month follow-up, 2099 participants (78.9%) completed the survey, of which 311 (14.8%) participants were found to still have drug use behavior in the past month. Drug use rates in MMT, MMT with CP and MMT with CM groups were 24.5%, 7.3% and 13.3%, respectively. The drug use rate in the MMT with CP group was significantly lower than that of MMT with CM (*x* ^2^  =  14.233; *P*<0.05), and the use rate in the MMT with CM group was significantly lower than MMT alone (*x* ^2^  =  27.824; *P*<0.05). Logistic regression analyses showed that both the MMT with CP group and the MMT with CM group were associated with lower drug use rates (*RR*  =  0.242 [0.174 – 0.337] and 0.475 [0.359 – 0.629], respectively). The injection rates in the MMT with CP and MMT with CM groups were significantly lower than that of MMT alone (5.1% and 6.9% vs. 16.3%; *x* ^2^  =  47.093 and 29.908, respectively; *P*<0.05). Additionally, logistic regression analyses showed a reduction in the follow-up injection rates in MMT with CP and MMT with CM groups compared with MMT (*RR*  =  0.267 [0.180 – 0.394] and 0.387 [0.270 – 0.555], respectively).

### Illicit drug use

Throughout treatment, 50165 urine tests were carried out in samples from 2662 participants, of which 7308 (14.6%) were positive. The urine test positive rate in the MMT with CP group was significantly lower than that of the MMT alone group (9.4% vs. 16.1%; *x* ^2^  =  340.848; *P*<0.05). However, the urine test positive rate in the MMT group was significantly lower than that of the MMT with CM group (16.1% vs. 19.4%; *x* ^2^  =  57.521; *P*<0.05). Among the 2662 participants, 2474 had at least one urine test. There were 457 participants with a positive result in the last urine test, demonstrating an illicit drug use rate of 18.5%. Illicit drug use in participants of the MMT with CP group was significantly lower than that found in participants in the MMT group (11.6% vs. 19.9%; *x* ^2^  =  22.220; *P*<0.05), and the rate in the MMT group was significantly lower than that in participants in the MMT with CM group (19.9% vs. 25.5%; *x* ^2^  =  6.628; *P*<0.05). Logistic regression analysis showed that the MMT with CP group was associated with lower illicit drug use rates (*RR*  =  0.534 [0.408 – 0.700]).

### Retention of MMT

**Figure 1:**
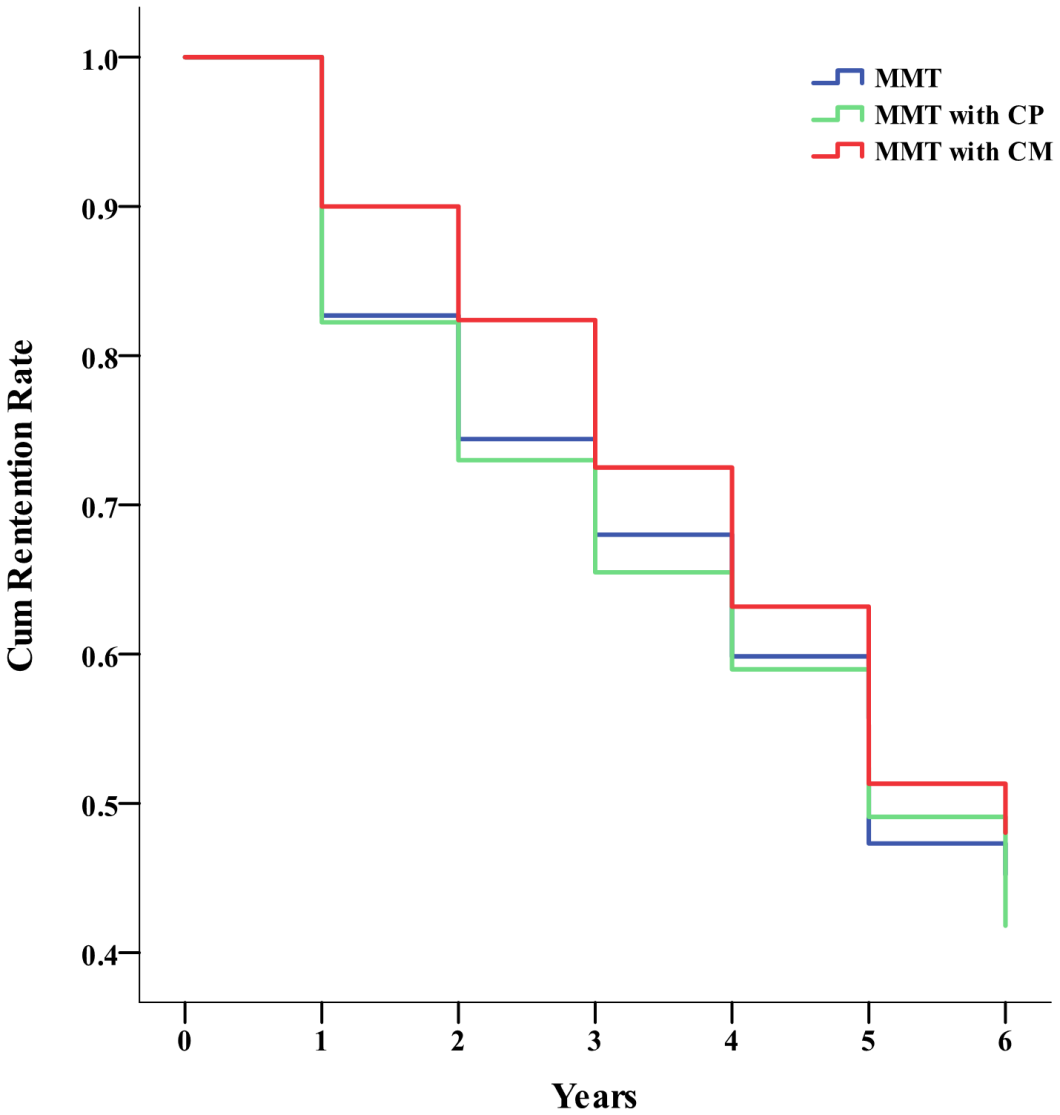
The cumulative retention rates of MMT participants from 2006 to 2011. The retention rate of participants in MMT with CM was significantly higher than that of MMT with CP (log-rank *x^2^*  =  7.490; *P*<0.05).

The average treatment retention time of all the 2662 participants was 1751 days, ranging from 3 days to 2009 days. The average retention time of participants of the MMT group was significantly shorter than that of the MMT with CP group (1744 vs. 1754 days; *Z*  =  –4.559; *P*<0.05) and the MMT with CM group (1744 vs. 1752 days; *Z*  =  –2.928; *P* <0.05). The first year retention rates of the three groups were 0.83, 0.82 and 0.90, with final retention rates of the three groups at 0.47, 0.49 and 0.51, respectively (Table 3). The retention rate of participants in the MMT with CM group was significantly higher than in the MMT with CP group (Log-rank *x^2^*  =  7.490; *P* <0.05) (Figure 1). Cox regression analyses of factors influencing the retention in MMT showed that in participants in the MMT with CM group, female gender, longer duration of drug use, other ethnicity, injection, and higher doses of drugs were associated with longer retention times (Table 4).

**Table 3:** Retention rates from 2006 to 2011.

Groups	Follow-up years	Subjects under Observation	No. of Drop-out	No. of Censoring	Retention Rate	*Z*	*p*
MMT	1	797	138	0	0.83	0.707 a	0.239
	2	659	66	0	0.74	0.447 a	0.326
	3	593	51	0	0.68	1.061 a	0.145
	4	542	65	0	0.60	0.354 a	0.363
	5	477	87	123	0.47	–0.707 a	0.239
MMT with CP	1	985	175	0	0.82	–5.657 b	<0.001
	2	810	91	0	0.73	–6.364 b	<0.001
	3	719	74	0	0.65	–2.828 b	0.002
	4	645	64	0	0.59	–1.414 b	0.079
	5	581	92	65	0.49	–0.707 b	0.239
MMT with CM	1	880	88	0	0.90	–4.950 c	<0.001
	2	792	67	0	0.82	–3.578 c	<0.001
	3	725	87	0	0.73	–1.768 c	0.038
	4	638	82	0	0.63	–1.061 c	0.145
	5	556	89	164	0.51	–1.414 c	0.079

a MMT vs. MMT with CP.

b MMT with CP vs. MMT with CM.

c MMT vs. MMT with CM.

**Table 4:** Multivariate Cox proportional hazards model for retention in MMT.

Variates	*B*	*SE*	*Wald*	*df*	*p*	*HR*	*95% CI*	
							***Lower Upper***
Groups			15.551	2	0.000			
MMT with CP	0.128	0.069	3.436	1	0.064	1.136	0.993	1.300
MMT with CM	–0.137	0.070	3.899	1	0.048	0.872	0.760	0.999
Gender	0.207	0.074	7.835	1	0.005	1.231	1.064	1.423
Years of drug use	–0.118	0.035	11.700	1	0.001	0.888	0.830	0.951
Ethnicity	–0.294	0.102	8.216	1	0.004	0.746	0.610	0.911
Injecting	–0.294	0.065	20.361	1	0.000	0.746	0.656	0.847
Dosage (mg)	–0.230	0.049	22.175	1	0.000	0.794	0.722	0.874

### HIV/HCV infection

There were 23 participants (0.9%) that tested positive for the HIV antibody. Eight cases showed seroconversion, with a incidence of 9.27 per ten thousand person-years during a follow-up of the 2639 negative anti-HIV participants. No seroconversion was found in participants in the MMT with CP group, while the HIV incidences in participants in the MMT and MMT with CM groups occurred at 20.09 and 10.02 per ten thousand person-years, showing that the HIV incidence in the MMT group was significantly higher than that of the MMT with CP group (*z*  =  2.513; *P*<0.05).

Two thousand four hundred ninety two of the 2662 participants received HCV tests, and 1642 (65.9%) of these were found to be antibody-positive. A total of 212 participants showed anti-HCV seroconversions with a incidence of 9.04 per hundred person-years during a follow-up of the 850 negative anti-HCV participants. HCV incidences of participants in the MMT, MMT with CP and MMT with CM groups were 18.35, 4.42 and 6.61 per hundred person-years, respectively. The incidence in the MMT group was significantly higher than in the MMT with CP group (*z*  =  8.464; *P*<0.05) and the MMT with CM group (*z*  =  6.423; *P*<0.05). Cox regression analysis showed that treatments from the MMT with CP and the MMT with CM groups were protective factors for HCV incidence (*RR*  =  0.209 [0.146 – 0.300], and 0.414 [0.298 – 0.574], respectively).

## Discussion

This study investigated and compared the short-term and long-term effects of MMT in conjunction with one of two different psychosocial intervention models. The results showed that MMT with CP or CM could reduce high-risk drug use behavior, with the drug use and injection rates of participants in the MMT with CP or CM group significantly lower than that of the MMT only group. Furthermore, the results from urine tests for the evaluation of illicit drug use during treatment showed that the MMT with CP group had the lowest rate of positive urine tests and illicit drug use, which is in agreement with results of a study by Gerrad *et al.* [35]. The highest rates were observed in the MMT with CM group, which contradicts some reports [29], [36], but is in agreement with a report by Epstein *et al.*, who similarly found that CM was less effective in reducing heroin use than cocaine use during MMT [37], and Saxon *et al.*, who reported that CM did not reduce the number of positive urine tests [38]. Studies by O'Brien *et al.* and Chutuape *et al.* indicated that behavioral interventions in CM would be more effective with the use of take-home or increased methadone doses among participants with positive urine tests [39], [40]. However, clinics in China used relatively low doses of methadone, and methadone was not allowed to take outside of clinics.

Treatment retention time is an important measure used in the evaluation of MMT effectiveness, with a longer retention time indicating a better therapeutic effect [41]. A meta-analysis of MMT participants in China reported that the retention rates at 12 and 24 months after enrollment were 55.2% and 43.0% [42]. Our results demonstrate higher retention rates at 12 and 24 months after enrollment in the five clinics in Xi’an of China. Previous studies have shown that CP and CM can improve the six-month retention rate in MMT [29], [30]. Our study shows that after a follow-up of almost six years, the retention time of participants in the MMT with CP and MMT with CM groups was significantly longer than that of the MMT only group. The retention rate of MMT with CM was significantly higher than that of MMT with CP. Cox regression analyses showed that in addition to gender, duration of drug use, ethnicity, injection of drug use and treatment dosage, the addition of different psychosocial intervention models is also an important factor for MMT retention. However, the difference of retention time among the groups was within 10 days and has no obvious clinical meaning. The relatively minimal difference may in part be due to the short duration of the psychosocial interventions. Hence, further studies are needed to examine the long-term effects of extended intervention periods.

Zhang *et al.* [43] reported that the national HIV prevalence in injecting drug users was 9.08% in 2010. Zhuang *et al*. [44] reported that the national HIV and HCV prevalence in the MMT clients was 6.0% and 60.1%, respectively. This study found that HIV and HCV prevalence in the participants was 0.9% and 65.9%, HIV prevalence was lower than both of the reports, but the HCV was higher than the results by Zhuang *et al.* [44]. Furthermore, we found that the HIV and HCV incidence in the CM and CP groups were significantly lower than that of the MMT only group. Zhang *et al.* [43] reported that the national HIV incidence in injecting drug users was 57 per ten thousand person-years, which was much higher than we found in MMT group (20.09 per ten thousand person-years). The HCV incidence in the MMT group (18.35 per hundred person-years) was close to the 20 per hundred person-years reported by Crofts *et al.* in Victoria [45], but was significantly higher than those of the MMT with CP and MMT with CM groups. The HCV incidences of the two groups were also lower than the 2 year’s follow-up results reported by Hagan *et al.* [46] in Washington (11.6 per hundred person-years).

Psychological practitioners provided the counseling in the two clinics employing CP intervention in this study. The implementation of targeted psychological counseling is of utmost importance considering the lack of counseling during treatment in China, in addition to the presence of misconceptions of MMT among some doctors and patients. The CM measures, such as material incentives for patients with better medication compliance and punishment for patients with positive urine tests, not only reduces the economic burden of medication for patients to a certain extent, but can also improve self-confidence and self-fulfillment in these patients [47]. A meta-analysis on CM by Griffith *et al.* showed that the effects of CM were correlated with intervention measures, intervention time, weekly urine test times, etc., and that additional CM measures, such as take-home incentives and increased methadone dosage, would provide a more robust effect [48].

This study utilized a large sample size of drug abuse participants, and included a long-term follow-up. In addition to drug use behavior and retention rates, this study also included HIV and HCV incidences as objective indicators for the evaluation of long-term effects of MMT. However, the study also had some limitations. Firstly, the participants were not randomly allocated to each of the treatment groups as a result of conditional limitations of the participating clinics and related ethical issues. Although randomized allocation was not carried out, comparisons of drug use and demographic characteristics, such as age, and gender, were found to be not significantly different among the three groups. Secondly, there were significant differences in methadone dosages in the different treatment groups. To control possible confounding factors, unconditional logistic regression models or Cox proportional hazard models were used to estimate the adjusted *RR*. On the basis of dosage adjusting, the results still showed that CP and CM were protective factors for high-risk drug use behavior and HCV incidence. Thirdly, monthly urine tests were performed instead of weekly tests, which could potentially lower the rate of positive urine tests obtained. Furthermore, psychological counseling could not be completely eliminated in participants in the MMT only group.

## Conclusions

This study compared the long-term effects of different psychosocial intervention models in participants at five MMT clinics in Xi’an of China, demonstrating that MMT supplemented with CP or CM can reduce heroin use and related risk behaviors, thereby reducing the incidence of HIV and HCV.
